# Evaluation of scaling-up of HPV self-collection offered by community health workers at home visits to increase screening among socially vulnerable under-screened women in Jujuy Province, Argentina

**DOI:** 10.1186/s13012-017-0548-1

**Published:** 2017-02-13

**Authors:** Silvina Arrossi, Melisa Paolino, Laura Thouyaret, Rosa Laudi, Alicia Campanera

**Affiliations:** 1 Centro de Estudios de Estado y Sociedad/Consejo Nacional de Investigaciones Científicas y Técnicas, Sánchez de Bustamante 27, Buenos Aires, 1193 Argentina; 2 Programa Nacional de Prevención de Cáncer Cervicouterino/Instituto Nacional del Cáncer, Julio A. Roca 781, Buenos Aires, 1067 Argentina; 3 Ministerio de Salud de la Provincia de Jujuy, Av. Italia esq. Independencia, San Salvador de Jujuy, 4600 Argentina

**Keywords:** Cervical cancer prevention, Self-collection HPV test, Argentina, Health System Framework, Implementation research, RE-AIM Framework, Scaling-up

## Abstract

**Background:**

Self-collection has been proposed as a strategy to increase cervical screening coverage among hard-to-reach women. However, evaluations of the implementation of this strategy on a large scale are scarce. This paper describes the process and measurement of the scaling-up of self-collection offered by community health workers during home visits as a strategy to reach under-screened women aged 30+ with public health coverage, defined as the target women.

**Methods:**

We used an adaptation of the Health System Framework to analyze key drivers of scaling-up. A content analysis approach was used to collect and analyze information from different sources. The RE-AIM (Reach, Effectiveness, Adoption, Implementation, and Maintenance) model was used to evaluate the impact of the strategy.

**Results:**

HPV self-collection was scaled-up in the province of Jujuy in 2014 after a RCT (Self-collection Modality Trial, initials EMA in Spanish) was carried out locally in 2012 and demonstrated effectiveness of the strategy to increase screening uptake. Facilitators of scaling-up were the organizational capacity of the provincial health system, sustainable funding for HPV testing, and local consensus about the value of the technology. Reach: In 2014, 9% (2983/33,245) of target women were screened through self-collection in the Jujuy public health sector. Effectiveness: In 2014, 17% (*n* = 5657/33,245) of target women were screened with any HPV test (self-collected and clinician-collected tests) vs. 11.7% (4579/38,981) in 2013, the pre-scaling-up period (*p* < 0.0001). Implementation: Training about the strategy was provided to 84.2% (*n* = 609/723) of total community health workers (CHWs). Of 414 HPV+ women, 77.5% (*n* = 320) had follow-up procedures. Of 113 women with positive triage, 66.4% (*n* = 75) had colposcopic diagnosis. Treatment was provided to 80.7% of CIN2+ women (*n* = 21/26). Adoption: Of trained CHWs, 69.3% (*n* = 422/609) had at least one woman with self-collection; 85.2% (*n* = 315/368) of CHWs who responded to an evaluation survey were satisfied with self-collection strategy. Maintenance: During 2015, 100.0% (723/723) CHWs were operational and 63.8% (461/723) had at least one woman with self-collection.

**Conclusions:**

The strategy was successfully scaled-up, with a high level of adoption among CHWs, which resulted in increased screening among socially vulnerable under-screened women.

**Electronic supplementary material:**

The online version of this article (doi:10.1186/s13012-017-0548-1) contains supplementary material, which is available to authorized users.

## Background

A key component of cervical cancer prevention programs is the achievement of high screening levels, particularly among poor women with low access to the health system. It is well known that human papillomavirus (HPV) testing has important advantages over the Pap as a screening test: high sensitivity (over 90%) [1] and high negative predictive value [2]. Very importantly, through self-collection, HPV testing can reduce barriers to screening and increase coverage [3], especially among hard-to-reach women who are at higher risk of cervical cancer [4]. The method is highly accurate [5], acceptable to women, and effective to increase screening uptake [6–10]. However, this evidence comes mainly from controlled research studies. Although in the last years there has been an increasing recognition of the importance of documentation and analysis of how scaling-up experiences are initiated, led, and monitored [11], very little evidence exists about how to scale-up HPV self-collection, potentially jeopardizing its successful integration into cervical cancer prevention programs.

In 2012, self-collection was implemented in the province of Jujuy as part of theEMA study (Self-collection Modality Trial, initials EMA in Spanish), a mix-method research study [9, 12, 13] that included a randomized controlled trial (RCT) to evaluate effectiveness of HPV self-collection offered by community health workers (CHWs) at home visits to increase screening uptake [9]. The intervention resulted in a fourfold increase in screening uptake (from 20.2% to 85.9%), demonstrating that offering the strategy was effective to improve cervical screening uptake [9]. Based on these findings, in 2014, self-collection of HPV testing was scaled-up to the whole province. The project was led by the National Cancer Institute, Argentina, in collaboration with the Jujuy Ministry of Health.

In this article, we report results of a study carried out to evaluate the scaling-up of HPV self-collection using implementation research methods. For this, we used an adaptation of the Health System Framework [14, 15] (HSF) and the Reach, Effectiveness, Adoption, Implementation, and Maintenance (RE-AIM) model for evaluation [16]. The specific aims of the study were to (1) identify key drivers of the scaling-up of HPV self-collection; (2) evaluate if self-collection was effective to increase screening uptake among under-screened women when implemented on a larger scale; (3) evaluate if the self-collection strategy was accepted and adopted by CHWs; and (4) identify main implementation barriers. Thus, the study provides evidence about the scaling-up process that is key for countries considering incorporation of HPV testing into their cervical cancer control programs.

## Methods

### Intervention context

A detailed description on the context can be found elsewhere [9, 17], but briefly in Argentina, the population not covered by the social security sector (workers of the informal economy and their families) has public health coverage; health care is provided free of cost. Jujuy is located in Northwest Argentina; 45.2% of population has public health coverage [18]. Its public health system includes a main hospital and 270 primary health care (PHC) centers.

The PHC system employs more than 700 full-time CHWs who visit around 110,000 households (70% of total provincial households) [18] twice yearly for health-related tasks including promotion of HPV testing at health centers. Non-visited households are mainly those located in middle-high and high income urban areas. During the visits, CHWs collect socio-demographic and health data of the household and its members, which is used to annually update the PHC census.

A national screening information system (SITAM) [19] registers data on screening tests and diagnostic procedures from women attending opportunistically public health centers, as there is no call and re-call system.

**Fig. 1:**
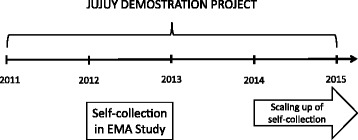
Timeline of the Jujuy Demonstration Project

Scaling-up of self-collection was nested into the Jujuy Demonstration Project (JDP) carried out between 2011 and 2014 (see Fig. 1) to evaluate the introduction of the HPV test as the primary screening method for women aged 30+ attending public health services [17]. Cytology is used as the triage test of HPV+ women.

### The intervention

In Jujuy, in 2014, the strategy of self-collection offered by CHWs during home visits was scaled-up for all women with public health coverage and without a screening test in the last 5 years (*n* = 33,245). To implement the strategy, health authorities decided to involve all 723 CHWs belonging to PHC system. The core components of the intervention scaled-up included the offer of self-collection during home visits by CHWs, sample handling and transportation, follow-up of HPV+ women and treatment if needed, and training of CHWs, as described below.

### Target population

Target population were all women aged 30+ included in the PHC census, not screened in the previous 5 years (under-screened women) with public health coverage.

#### Offer of self-collection

Self-collection was offered by CHWs during home visits throughout the second half of 2014. During the visit, CHWs provided women with information about cervical cancer prevention and HPV testing and then offered them HPV self-collection, followed by a 10-min step-by-step explanation on how to perform it using communication support material (Additional file 1). Self-samples were collected using the Qiagen cervical sampler during the CHW visit. Pregnant women were not offered self-collection.

#### Sample handling and transportation to PHC centers

CHWs labeled collectors with the woman name and the national unique identifier number and transported specimens at room temperature to PHC centers: from here they were sent to the provincial HPV laboratory by the health center. Samples were tested for 13 high-risk HPV types using hybrid-capture 2, following manufacturer’s instructions. Specimens were not processed if received >14 days after collection, without liquid, brush, or identification data (discarded samples).

#### Follow-up and treatment

According to guidelines [20], self-collected HPV+ women were referred to cytology triage. HPV+ women with normal cytology were advised to repeat test at 18 months. Women with abnormal cytology (atypical squamous cells of undetermined significance; atypical cells cannot rule out high-grade squamous intraepithelial lesion; high-grade squamous intraepithelial lesions; and cancer) were referred to colposcopy, and biopsy if needed. Biopsies were reported according to cervical intraepithelial neoplasia (CIN) terminology. Identified cases of CIN2+ were treated according to standard protocols (loop electrosurgical excision procedure (LEEP) for CIN). HPV-negative women were advised to repeat screening within 5 years following national recommendations.

#### Training of CHWs

From April to June 2014, national and provincial team members led 18 workshops aimed at training of all 723 CHWs. Description of structure and content of workshops are shown in Table 1. They included expert presentations, discussions in small groups, and role playing to recreate different scenarios during the offer of self-collection. At the end of each training workshop, CHWs were provided with the list of women to be offered the test, self-collection kits, and other materials needed for the offer (i.e., educational materials). If needed, additional collectors/materials could be requested to health centers or the provincial program headquarters.

**Table 1:** Description of training workshops

Sections	Contents
Project background	Information on the EMA study and scaling-up in a programmatic context
Cervical cancer	Scientific data on cervical cancer and its relation with HPV
HPV testing	Basic information on HPV testing as primary screening strategy for cervical cancer prevention
HPV self-collection	HPV self-collection: clinician vs. self-collected tests, step-by-step self-collection take-up, self-collection results understanding, and follow-up of HPV+ women
Communication skills	Communication skills to conduct the educational talk (instruct women on how to perform self-collection)
Logistical procedures	List of women to be offered self-collection, collectors, and training on sample labeling and transportation

By the end of 2014, once the round of home visits was finished, an evaluation workshop was carried out with all CHWs. During this activity, CHWs were invited to complete a self-administered questionnaire (SEAQ) to elicit their views and opinions about the incorporation of HPV self-collection as a programmatic activity. At the beginning of 2015, a refresher training was provided to all CHWs.

### Evaluation of scaling-up

To answer the different questions addressed by the study, we used a combination of methods:

1. Analysis of the scaling-up process. For this analysis, we used an adaptation of the HSF [14, 15], which includes contextual interconnected factors that are considered key drivers for successful scaling-up [21]. The HSF integrates six functions, or building blocks (service delivery, health workforce, information, technology, funding, stewardship) that influence system performance. We have re-configured and simplified these functions to incorporate an organizational dimension, which is a key factor for cervical cancer prevention program effectiveness [22]. Thus, this adaptation presents four dimensions for analysis, instead of the original six, as service delivery, health care workforce, and information have been integrated into the dimension organizational capacity, as follows (Fig. 2).

**Fig. 2:**
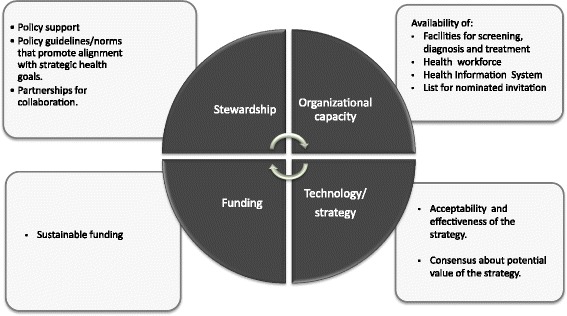
Health System Framework for analysis of cervical cancer prevention
  - Stewardship: Policy support; existence of policy guidelines/norms that promote alignment with strategic health goals; partnerships for collaboration
  - Organizational capacity: Capacity of the health system to implement the intervention in all stages of the screening/diagnoses/treatment continuum, including facilities and health workforce; and availability of health information systems
  - Technology/strategy: Acceptability, effectiveness, and consensus about the value of the technology/strategy
  - Funding: availability of sustainable funding
  The analysis was based on content analysis of program reports (information sheets, power point presentations, etc.) and observation derived from our participation in the policy definition. The analysis was verified by triangulation across the different sources. Also, preliminary results were presented and discussed in a meeting of provincial policy makers and health providers, as well as in the final evaluation workshop, to discuss and verify research findings.
2. Analyses of dimensions proposed by the RE-AIM model [16, 23].

#### Reach

We measured the percentage of under-screened women with public health coverage with self-collected tests/total target population. Additionally, we compared target women with HPV self-collection and the total target population according to their age distribution using simple frequencies and percentages.

#### Effectiveness

We evaluated two outcomes: (1) The increase in screening uptake among the target population. For this, we compared the difference between the percentage of the target population who was HPV-tested (both with self-collection and clinician-collected tests at health centers)/total target women in 2014 (post scale-up) and the percentage of the target population screened at health centers/total target women in 2013 (the year previous to self-collection introduction). (2) The increase in the proportion of total screened women who were from the target population. For this, we compared the pre and post scaling-up percentage of screened women who were from the target population/total screened women. Pre and post scaling-up percentages were compared using chi-square tests of association, and statistical significance was established at *p* < 0.05.

#### Adoption

Three outcomes were measured: (1) percentage of adopters: number of CHWs with at least one self-collected sample registered in SITAM/total trained CHWs; (2) average number of women with self-collected tests per CHW; and (3) percentage of CHWs satisfied with the self-collection strategy/total CHWs who answered the SEAQ.

#### Implementation

Three outcomes were measured: (1) percentage of trained CHWs/total CHWs; (2) percentage of CHWs who reported at least one problem to obtain materials (kits, leaflets)/total CHWs who answered the SEAQ; and (3) percentage of discarded samples registered in lab records/total number of self-collected tests.

Additionally, for this dimension, we analyzed follow-up in the target population: (1) percentage of HPV+ women with follow-up procedures, HPV+ women were considered to have received follow-up if they had at least one of the following procedures, independently of the protocol recommendation: cytology triage, colposcopy, or second HPV negative test; (2) percentage of HPV+ women with cytology triage (as per protocol); (3) percentage of women with positive triage who had colposcopic diagnosis/total women with positive triage; and (4) percentage of treated women/women with CIN2+ lesions.

#### Maintenance

One outcome was measured: percentage of trained CHWs with at least one self-collected sample registered in SITAM in 2015/total trained CHWs.

### Databases

Three databases were built specifically for the study. First, we linked the 2013 PHC census to the SITAM database to draw up the list of target women to be offered self-collection. After field work, we linked the list of target women to SITAM to extract data on HPV testing, triage, diagnosis, and treatment, using each woman’s national identity document number. To calculate the percentage of screening uptake in 2013, we linked the 2012 list of target population to the SITAM database. We also built a specific database with CHWs information, including sex, type of setting, and participation in training workshops.

Finally, we used the SEAQ to elicit CHWs views about the offer of self-collection, specifically on their satisfaction with the strategy, and problems during the implementation. CHWs were asked to fill the SEAQ during the final evaluation workshop carried out at the end of the health round. It was anonymous, and participation was voluntary; a member of the research team explained its objectives and how to complete it.

## Results

Results of the analysis based on the Health System Framework are presented below.

### Stewardship

The EMA study was a research project carried out collaboratively by national and provincial Ministries of Health (MoHs). Expansion of the strategy was discussed by the National Program of Cervical Cancer Prevention (NPCC) with provincial authorities by the end of 2013 after results from the EMA study were available. Scaling-up was considered key to reach provincial screening coverage objectives [17]. A proposal was presented to and approved by the Jujuy Demonstration Project Scientific Advisory Committee (Advisory Committee), which included members from main medical scientific societies, international health organizations (Pan American Health Organization, International Agency for Research on Cancer, and United Nations Population Fund), and women organizations such as the Women National Council. Thus, the decision to scale-up was agreed in a context of high support by national and provincial health authorities, and international health organizations.

Next, core components and algorithm of the scaling-up (described above) were defined on a national/provincial management round-table. Their members met at least twice during the implementation phase to monitor and evaluate process results, as well as to identify emerging problems. Introduction of HPV self-collection was incorporated into provincial norms and regulations, and national protocols [20].

### Organizational capacity

The organizational capacity of the province was considered adequate. Jujuy has a wide network of PHCs, and CHWs visit houses twice a year as a regular, programmatic activity. A referral network for triage, diagnosis, and treatment was already in place, and health providers had received training on HPV testing in the context of the JDP initiated in 2011 [17]. The province runs the first country public HPV laboratory, with more than 50,000 samples processed between 2012 and 2014. A team of two navigators identifies women with abnormal Pap smears to provide support in cases where women have difficulties to complete diagnosis/treatment. However, no specific support is provided to HPV+ women who need to be triaged. SITAM, the information system for monitoring and evaluating screening activities [19], was already in place, with registry of all women being screened at public health institutions. SITAM was used to build the nominated list of target women to be offered self-collection by CHWs.

### Technology/strategy

Local evidence indicated that the strategy was both acceptable to women [9, 12] and CHWs [13], and effective at increasing screening uptake [9], in agreement with studies carried out in other countries and settings. Local qualitative research carried out by our team had also shown its high acceptability among provincial health professionals, especially as a strategy to reach under-screened women (Arrossi, unpublished observations). The Advisory Committee also acknowledged the strong potential of HPV self-collection to increase uptake although their members expressed concern about its relatively lower detection rate when compared to clinician-collected tests [5]. As a result, the Advisory Committee strongly recommended HPV self-collection to screen hard-to-reach women. The Advisory Committee also discussed and agreed on the proposed algorithm for HPV self-collection, which implied a modification of the algorithm used in the EMA RCT [9]. In effect, due to the relatively low performance of colposcopic diagnoses in the absence of a Pap smear result found in that project (Arrossi, unpublished observations), it was decided that HPV+ women would be referred to cytology triage.

### Funding of scaling-up

Funding of the scaling-up was evaluated as sustainable, as it was implemented in the context of the JDP, with funding by the national and provincial MoHs. The HPV test was provided by the national MoH as part of the JDP. In addition, the EMA study was carried out in a programmatic context, with provincial health providers and infrastructure funded through the regular budget. Therefore, a decision was made by health authorities to guarantee the funding needed to scale-up the strategy. Triage, diagnosis, and treatment of HPV+ women were provided by public health institutions, free of cost for women, using the provincial referral network, which has been described elsewhere [17]. Communication materials used for the offer of self-collection were those used in the EMA RCT and provided to the province by the NPCC, as part of the regular provision of communication materials by the national MoH to provinces. SITAM is run and maintained by the national MoH.

### RE-AIM measurement

#### Reach

In 2014, 2983 women from the target population (9%) were screened with self-collected tests. Compared to the target population (Table 2), more women with self-collected tests were aged 35–44 (37.0 vs. 32.8%) and less women were aged 65+ (4.6 vs. 8.7%).

**Table 2:** REACH: Comparison of women with self-collected tests with total target population, 2014

	Target population with self-collected test		Total target population		*p* value
	*n*	%	*n*	%	
Age					
30–34	677	22.8	7827	23.5	*p* < 0.001
35–44	1105	37.0	10,908	32.8	
45–54	615	20.6	6580	19.8	
55–64	448	15.0	5033	15.2	
65+	138	4.6	2897	8.7	
Total	2983	100.0	33,245	100.0	

*Chi-square test, statistical significance was established at *p* < 0.05

#### Effectiveness

In 2014, 12,778 women aged 30+ were HPV-tested in the Jujuy public health sector, 44.3% (*n* = 5657/12,778) were target population, a 38% increase from 2013 (32.1%; *n* = 4579/14,272; *p* < 0.0001) (Table 3). Of the target population, 17.0% (*n* = 5657/33,245) were HPV-tested with any HPV test (2983 with self-collected tests and 2674 with clinician-collected tests at health centers). This is a 45% increase in screening uptake of the target population relative to pre scale-up level in 2013 with clinician-collected tests at health centers (11.7%; *n* = 4579/38,981; *p* < 0.0001).

**Table 3:** RE-AIM measurement

RE-AIM dimension	Question	Outcome	Value
Reach	What are the characteristics of women reached by the strategy?	Under-screened women with public health coverage with self-collected test (2014)	9.0%
Effectiveness	Is the strategy effective to increase screening uptake in target population?	Screened women from target population, post-intervention (2014)	44.3%
		Screened women from target population, pre-intervention (2013)	32.1%
		Target population with screening uptake, post-intervention (2014)	17.0%
		Target population with screening uptake, pre-intervention (2013)	11.7%
Adoption	Is the self-collection strategy accepted and adopted by CHWs?	CHWs with at least one self-collected test registered in SITAM	69.3%
		CHWs with at least one self-collected test in target population registered in SITAM	62.6%
		CHWs that mentioned that they were satisfied with self-collection strategy	85.6%
Implementation	To what extent the intervention was implemented as intended?	Trained CHWs/total CHWs	84.2%
		CHWs who reported at least one problem to obtain materials	20.7%
		Discarded samples registered in lab records/total number of self-collected tests	0.9%
		Follow-up in TP	
		HPV+ with follow-up procedures	77.5%
		Triage + women with colposcopy	66.4%
		Women with CIN2+ with registered treatment	80.7%
Maintenance (1 year)	Is it possible to sustain the intervention over time?	Trained CHWs with at least one self-collected test registered in SITAM in 2015	63.8%

*TP* target population, *CHWs* community health workers

#### Adoption

In total, 609/723 (84.2%) CHWs were trained; 69.3% (*n* = 422/609) of them had at least one woman with a self-collected sample registered in SITAM; 62.6% (*n* = 381/609) if only women from the target population are considered (Table 3). The average number of women with self-collected samples per CHW was 11 (range 1–74), and eight if only screening in the target population is considered (range 1–31).

Of the 368 CHWs who completed the SEAQ, 85.6% of (*n* = 315) mentioned that they were satisfied with the self-collection strategy (Table 3).

#### Implementation

Training about the strategy was provided to 84.2% (*n* = 609) of total CHWs (Table 3). When asked about problems during implementation, 20.7% reported having at least one problem to obtain collectors, leaflets, or materials needed for the offer. Percentage of discarded samples at the laboratory was 0.9% (29/2983).

#### Adherence to follow-up, diagnosis, and treatment

**Fig. 3:**
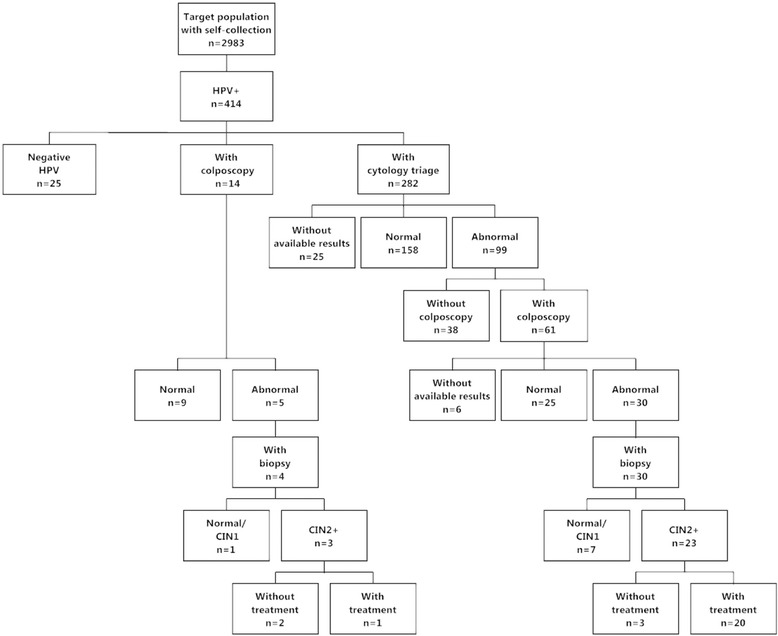
Follow-up of HPV+ women among target population. Jujuy 2014

Of the 2983 target women with self-collected tests, 414 (13.9%) were HPV+ (Fig. 3). Of these, 77.5% (*n* = 321) had follow-up procedures: 282 (68.1%) had cytology triage, as per protocol: 158 were normal, 99 abnormal and there were no available results for 25; 14 had only colposcopy, and 25 had a second negative HPV test. Total women with colposcopic diagnosis were 75 (66.4% of women with positive triage), including 14 colposcopies without cytology result (34 normal, 35 abnormal; 6 women had colposcopies at private services without available results).

Among women with abnormal colposcopy, 34 had biopsies performed at public health services and had results in SITAM. Among them, 26 had CIN2+ (detection rate 0.9%, *n* = 26/2983); 21 of these (80.7%) received treatment (follow-up until June 30, 2016).

#### Maintenance

In the second year of the scaling-up process (2015), 723 (100.0%) CHWs were operational: all 609 CHWs trained in 2014 and 114 incorporated and trained in 2015. In that year, 63.8% (461/723) had at least one woman with a self-collected test registered in SITAM (Table 3).

## Discussion

Increasing screening uptake among hard-to-reach women is a major critical factor for cervical cancer control. Using the RE-AIM model and the HSF, we analyzed the scaling-up of HPV self-collection offered by CHWs at home visits, in the province of Jujuy, Argentina. The strategy resulted in a 45% increase in screening of under-screened, socially vulnerable population. Results also showed that most CHWs adopted the strategy, with few implementation problems. Leadership and partnership of national and local health decision makers, governmental funding, an adequate organizational capacity of the health system, and consensus about the potential value of the strategy were identified as key drivers of the scaling-up.

This is, to our knowledge, the first study analyzing scaling-up of a HPV self-collection strategy using implementation science methods. Utilization of the RE-AIM framework allowed us to include in the analysis the five factors that more completely characterize the public health impact of an intervention carried out in real-world environments [16]. In addition, the HSR framework guided the evaluation to identify key drivers of the scaling-up process. Thus, this study can serve as a model of how implementation science can be used to plan, conduct, and evaluate interventions that are implemented on a large scale in programmatic, real-world contexts. Stewardship refers to the policy environment in which the implementation of a health strategy is made possible, through three main dimensions: formulating health policy, exerting influence, and collecting and using intelligence [24]. In our study, scaling-up of HPV self-collection as part of a national demonstration project to introduce HPV testing as primary screening [17] provided governmental stewardship in a context of high policy support. This was reinforced by involvement of the scientific community and other main health and women organizations, which ensured alignment of objectives and goals. Another key factor was that scaling-up was based on the EMA study, a research project carried out in a programmatic context and run collaboratively between the national and provincial health authorities. Incorporating research into implementation, country ownership, and political will have been identified as factors associated with faster diffusion of an innovation [25, 26]. By making use of legal, regulatory, and policy instruments to steer health system performance [24], stewardship bears a strong relationship to the concept of regulation [27]. In our study, inclusion of the strategy into national and provincial norms and protocols was also identified as a key enhancer of the scaling-up.

Integrating activities into existing health systems has been identified in the literature as an important factor for successful scaling-up [28]. In this case, expansion followed the EMA study, using the provincial organizational capacity. Health system weaknesses contributing to poor health outcomes include poor management and a weak PHC system [29]. In Jujuy, the PHC system is well developed, with almost 700 CHWs visiting houses twice a year. The health system included the network of diagnosis and treatment units and the first public HPV laboratory of the country. Lack of information systems is also a key problem affecting cancer control programs [30]. The use of SITAM allowed not only to closely monitor and evaluate the scaling-up but also to build the target population list, facilitating identification and contact with under-screened women.

Securing sustainable funding for large-scale implementation can be a major challenge. Many pilot or research studies are funded by non-governmental national or international organizations, which results in funding very often ending with the pilot study [31]. Also, interventions proved efficacious in research studies carried out using industry-donated technology might be difficult to scale-up once the research is finished [32]. All funding for this project was provided by the regular mechanisms of public health system financing. Thus, scaling-up was guaranteed and stewardship strengthened, sending also a strong message to the community about the government commitment with its implementation in the context of the national policy for cervical cancer prevention.

The study showed increased screening among hard-to-reach women compared to what was attained the year previous to the scaling-up. It is possible that part of the population tested with clinician-collected tests were women offered self-collection by CHWs who preferred to get screened at health centers. This was observed in the RCT of the EMA study, where 15% of women contacted by CHWs preferred clinician-collected tests [9]. However, our study design did not allow measuring the impact of offering HPV self-collection on screening performed at health centers. It is neither possible to know if women reached by HPV self-collection would have eventually been screened at health centers, but in any case, the strategy is at least advancing the date of screening of these women. It is possible as well that the women reached by self-collection would have never been screened at all. Evidence shows that under-screened women have a higher risk of developing cervical cancer [33]. In addition, women with public health coverage are a socially vulnerable population, with over-representation among women with cervical cancer in Argentina [34]. If the strategy is maintained, and its pace, 80% of these hard-to-reach, high-risk women, could be screened in 5 years with high potential impact on cervical cancer control among this population.

Adoption has been described as the intention to employ an innovation or evidence-based practice [35]. Our study showed an adequate level of adoption of the strategy by CHWs, with 69% of trained CHWs with a self-collected test registered in SITAM. This is probably an under-estimation of the level of adoption as some CHWs might have adopted the strategy without being successful in the offer of self-collection, due to women characteristics or circumstances beyond his/her control. That is also a possible explanation to the fact that when we analyze the percentage of CHWs with at least one self-collected sample in the target population, the figure drops to 62%. Most CHWs were satisfied with offering the strategy. A qualitative study on CHWs’ experiences about HPV self-collection [13] showed that the possibility of having an active role in cervical cancer prevention activities was a main motivation factor for CHWs.

The implementation dimension in the RE-AIM model refers to the extent to which a program is delivered as intended [16]. In our study, training was delivered to the vast majority of CHWs, and there were almost no discarded samples at the laboratory. Few problems were reported with provision of self-collection materials. Availability of these supplies has a direct impact on the possibility of offering the test, and therefore, an interruption in their provision must be closely monitored. Finding a low frequency of reported issues was considered particularly important, as problems in access to inputs essential to CHWs provision of health services can negatively affect their work [36].

High coverage will not result in a decrease of disease burden if women are not diagnosed/treated. The protocol for scaling-up of self-collection recommended cytology as the triage test. Still there were 14 HPV+ women who had colposcopy without a previous cytology, revealing a problem of adherence to the triage protocol which has also been found in another study [3]. In our study, 77.5% of HPV+ women had follow-up procedures. Compliance with triage is lower than in the EMA RCT [9] and in a Chilean study [7] where 86% of women had colposcopic referral after an HPV positive test. However, the scaling-up protocol included a visit for cytology triage, an additional step not included in the EMA study. In a study carried out in France among non-attenders of low socio-economic level, 41% of women had a Pap smear after a positive HPV self-collected test [37]. Adding a visit to under-screened women poses a problem, as very probably the barriers faced by women to get screened at health centers [38, 39] partly account for the loss to cytology triage. This underscores a major limitation of self-collection followed by cytology. The protocol in place at health centers for clinician-collected samples states that HPV tests and Pap smears must be taken at the same time [17], but this is not possible in a self-collection context. Among women with self-collected tests, CIN2+ detection rate was 0.9%. This rate is lower than the one reported in the literature [5], and specifically in the EMA RCT [9], most probably due to the loss to follow-up, both at triage and colposcopic diagnosis.

Our study has a main limitation. We were not able to measure how many women were contacted by CHWs; therefore, we cannot evaluate how successful they were in the offer of self-collection. When we compare the average number of self-collected samples per CWH reported in the EMA RCT with the average obtained during scaling-up of the strategy, we observe that the figure drops (11 vs. 30). This decrease can be explained by several factors. On the one hand, the EMA study included only CHWs with good evaluation scores, whereas scaling-up was implemented with incorporation of all CHWs, independent of their performance scores. Including less-motivated CHWs might have had an impact in the strategy. Also, in the EMA RCT, women at home during the CHW visit were offered self-collection irrespective of their past Pap screening history, whereas in the scaling-up, CHWs were asked to offer self-collection to under-screened women with public health coverage. Women who were not at home during the CHW visit could not be offered the strategy, as CHWs visit houses once per round. Besides, the question on satisfaction in the SEAQ was only answered by 61% of CHWs; therefore, the reported level of satisfaction could be biased.

## Conclusions

We showed that HPV self-collection offered by CHWs at home visits can be adequately scaled-up in programmatic conditions to increase screening of hard-to-reach women. The strategy had a high level of adoption among CHWs, and there were few implementation problems at the screening phase, but obstacles for follow-up and treatment. It is important to devise new strategies and tests to increase triage and diagnosis among HPV+ women. This study provides key evidence for countries and programs planning to incorporate and expand HPV self-collection.

## Additional file

Additional file 1: Communication support material explaining all steps of self-collection take-up. (PDF 1379 kb)

## Abbreviations

CHWs: Community health workers
CIN: Cervical intraepithelial neoplasia
HPV: Human papillomavirus
JDP: Jujuy demonstration project
LEEP: Loop electrosurgical excision procedure
MoH: Ministry of Health
NPCC: National Program of Cervical Cancer Prevention
PHC: Primary Health Care
RCT: Randomized control trial
SITAM: National screening information system
TP: Target population
