# Supplementary material for: Evaluation of scaling-up of HPV self-collection offered by community health workers at home visits to increase screening among socially vulnerable under-screened women in Jujuy Province, Argentina
Source: Implement Sci. 2017 Feb 13;12:17. doi: 10.1186/s13012-017-0548-1 (PMC5307871; doi:10.1186/s13012-017-0548-1)

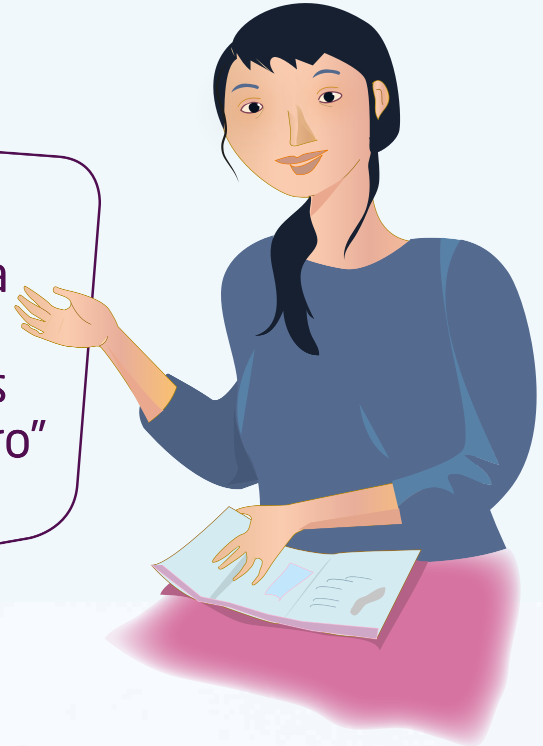

"Hacerte
vos misma
el test
de VPH es
fácil y seguro"

"Tubo colector y cepillo para la autotoma"

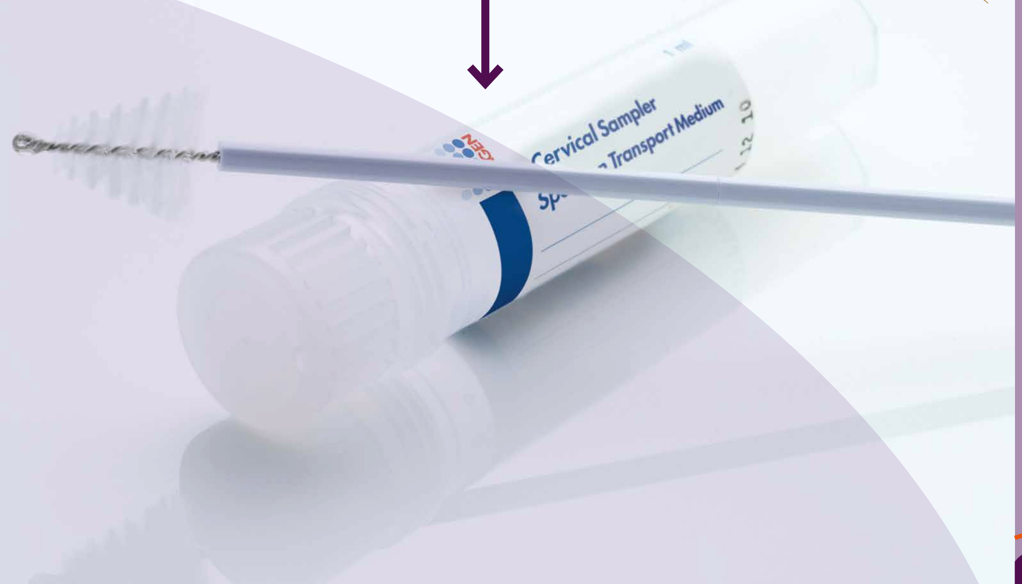

El cáncer de cuello de útero
SE PUEDE PREVENIR

Si sos mujer y tenés 30 años o más,
podes hacerte el Test de VPH

Es tu
derecho
ES TU
SALUD

0800 333 3586 - www.msal.gob.ar/inc

INC INSTITUTO NACIONAL del CANCER

Ministerio de Salud
Presidencia de la Nación

TEST DE VPH

"Ahora me puedo hacer
el test de VPH
yo misma"

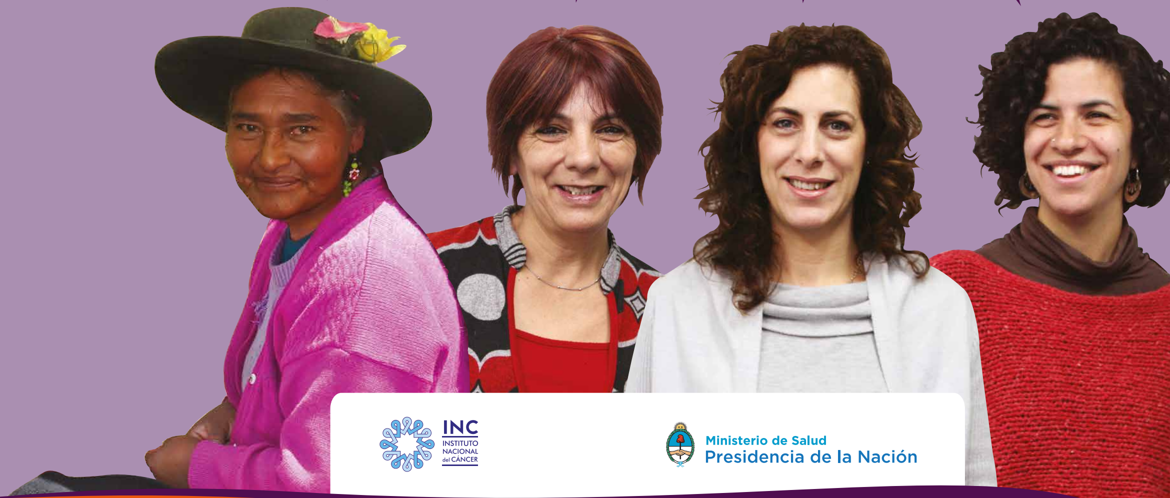

INC INSTITUTO NACIONAL del CANCER

Ministerio de Salud
Presidencia de la Nación

"Seguí los siguientes pasos"

1 Elegí el lugar de tu casa donde puedas tener privacidad.

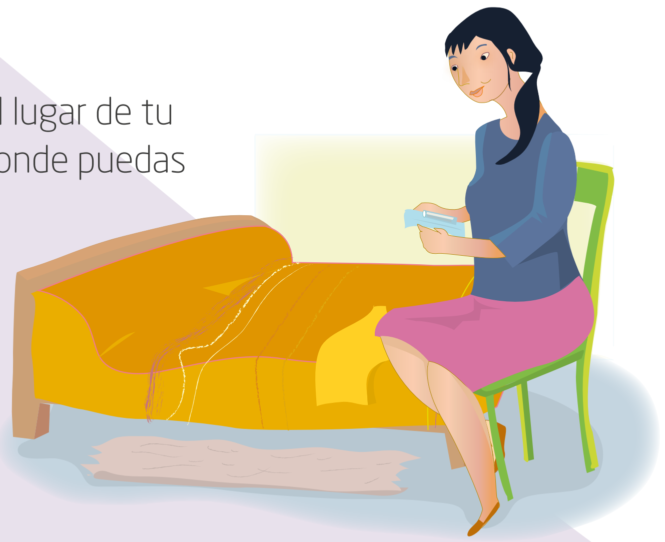

2 Buscá una posición que te quede cómoda (puede ser en cuclillas, parada, acostada sobre una cama con las piernas flexionadas o con una pierna arriba de una silla)

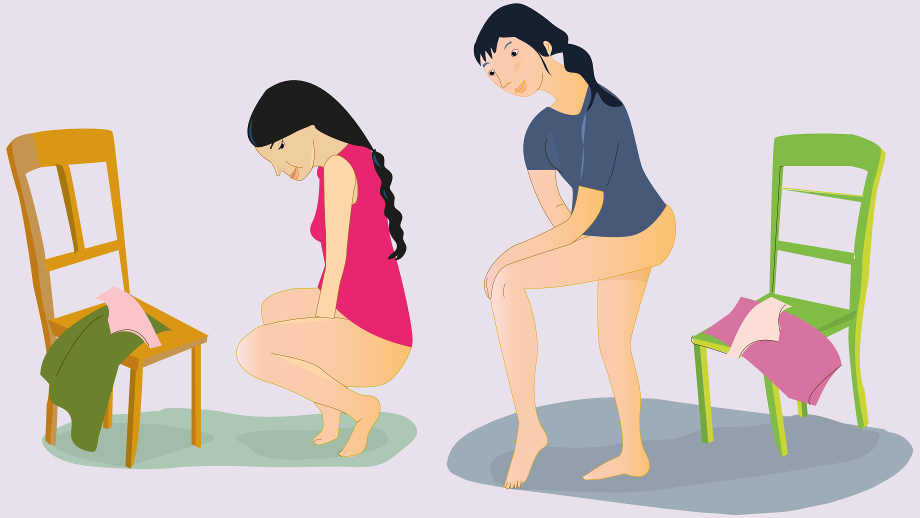

3 Con las manos limpias y luego de sacarte la bombacha, destapá el tubo y apoyá la tapa boca arriba. Es muy importante que lo tengas parado así no se derrama el líquido que tiene adentro

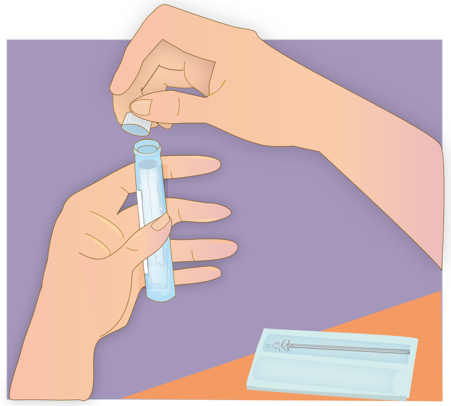

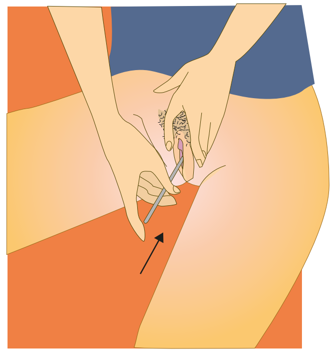

4 Tomá el cepillo con las manos y metelo en la vagina hasta que haga tope. Aproximadamente lo estarías introduciendo 10cm

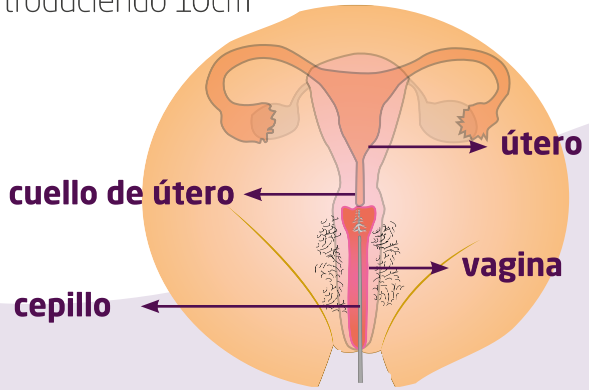

5 Una vez que el cepillo esté adentro, giralo 3 veces y sacalo de la vagina

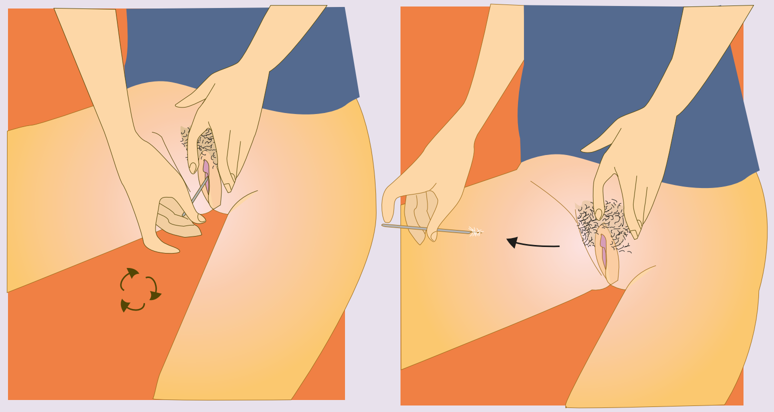

6 Meté el cepillo adentro del tubo, quebralo por la parte marcada así podés taparlo

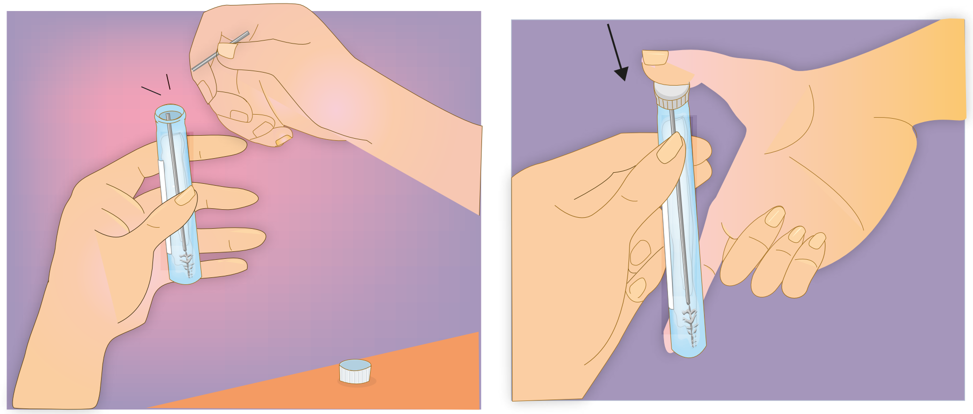

7 Entregalo a tu agente sanitario para que lo lleve a analizar al laboratorio de VPH

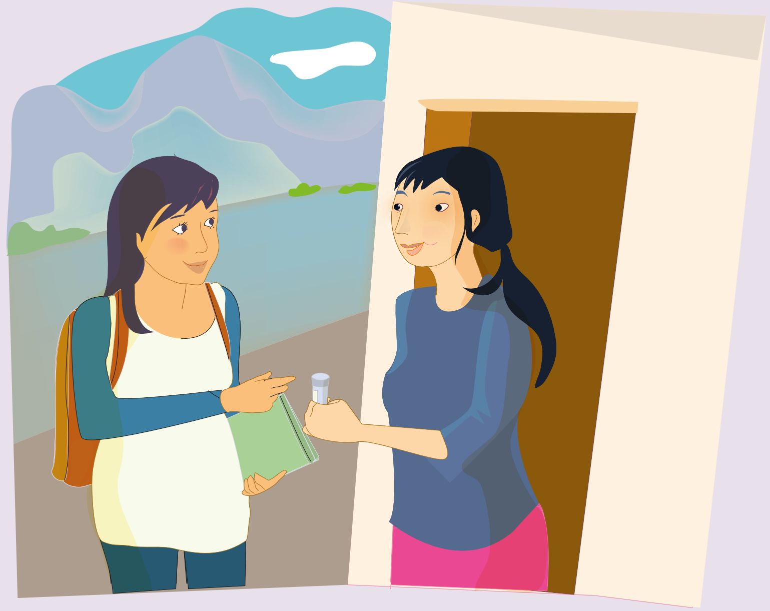

Supplement: Additional file 1: — Communication support material explaining all steps of self-collection take-up. (PDF 1379 kb) [file 13012_2017_548_MOESM1_ESM.pdf]
